# Requirement of *Pax6* for the integration of guidance cues in cell migration

**DOI:** 10.1098/rsos.170625

**Published:** 2017-10-04

**Authors:** Miguel Arocena, Ann M. Rajnicek, Jon Martin Collinson

**Affiliations:** 1 Institute of Medical Sciences, University of Aberdeen, Aberdeen, UK; 2 Facultad de Ciencias, UDELAR, Montevideo, Uruguay

**Keywords:** electrotaxis, contact guidance, neural progenitor, *Pax6*

## Abstract

The intricate patterns of cell migration that are found throughout development are generated through a vast array of guidance cues. Responding integratively to distinct, often conflicting, migratory signals is probably crucial for cells to reach their correct destination. *Pax6* is a master transcription factor with key roles in neural development that include the control of cell migration. In this study, we have investigated the ability of cells derived from cortical neurospheres from wild-type (WT) and *Pax6^−/−^* mouse embryos to integrate diverging guidance cues. We used two different cues, either separately or in combination: substratum nanogrooves to induce contact guidance, and electric fields (EFs) to induce electrotaxis. In the absence of an EF, both WT and *Pax6^−/−^* cells aligned and migrated parallel to grooves, and on a flat substrate both showed marked electrotaxis towards the cathode. When an EF was applied in a perpendicular orientation to grooves, WT cells responded significantly to both cues, migrating in highly oblique trajectories in the general direction of the cathode. However, *Pax6^−/−^* cells had an impaired response to both cues simultaneously. Our results demonstrate that these neurosphere derived cells have the capacity to integrate diverging guidance cues, which requires *Pax6* function.

## 1. Introduction

The integration of multiple, diverging guidance cues is likely to be a key feature of cell migration *in vivo*, as cells have to respond for instance to gradients of soluble attractant molecules as well to the adhesive and topographical features of the surrounding extracellular matrix and neighbouring cells [1]. In the nervous system, cell migration can be guided *in vivo* by growth factors and chemokines, and possibly also endogenous electric fields (EFs) [2–4]. During neural development, cells have also been observed to migrate along processes of neighbouring cells, indicating that they use topographical features of their microenvironment as guidance cues [5]. A deeper understanding of how these different guidance cues are integrated is likely to contribute to improve brain repair therapies that rely on neural stem cell migration, as well as being central to elucidating the regulation of cell migration during neural development.

The migration of cells using the topography of their microenvironment as guidance has been termed contact guidance, a phenomenon which has often been observed in the form of cells migrating along ordered fibres, either formed by extracellular matrix components or by processes from other cells [6]. Similarly, cells align on grooves engineered on the substrate and migrate along them [7]. EFs constitute a strong guidance cue, which can orient cell migration parallel to the EF vector, either towards the anode or the cathode, depending on the cell type, in a process termed electrotaxis [3]. In particular, neural stem cells display marked electrotactic responses, migrating with high directionality towards the cathode in an EF [8,9].

The combination of a grooved substrate and applied EFs of varying orientations has been developed as an effective means to expose cells to conflicting guidance cues, and it has previously been used to study the integrative capacities of corneal epithelial cells [10,11]. When the EF vector was oriented horizontally and grooves were oriented vertically, corneal epithelial cells showed an intermediate response between contact guidance and electrotaxis, that is, cells moved generally to the cathode, but with trajectories following a more vertical trajectory than in the absence of grooves [10]. This behaviour can be interpreted as a consequence of the integration of both guidance cues.

Neural precursors execute intricate patterns of migration during neural development, defects in which underlie several human neurological disorders [12]. The transcription factor *Pax6* has highly conserved roles in the development of the eye and the central nervous system [13,14] and it has been linked to the control of neural precursor migration during cortical development [15,16]. Also, neurons from Pax6 deficient embryos cultured *in vitro* showed defective polarization and impaired ability to migrate along pre-existent neurite bundles [17].

In this study, we have investigated the behaviour of wild-type (WT) and *Pax6^−/−^* cells derived from cortical neurospheres under contact guidance and EF cues. We have shown that, while *Pax6* is not required for the response to migratory signals in isolation, it is essential for the capacity of cells to integrate diverging guidance cues, which underlies the establishment of the appropriate patterns of cell migration during development.

## 2. Results

### 2.1. Response of cells derived from cortical neurospheres to contact guidance cues and electric fields in isolation

**Figure 1.**
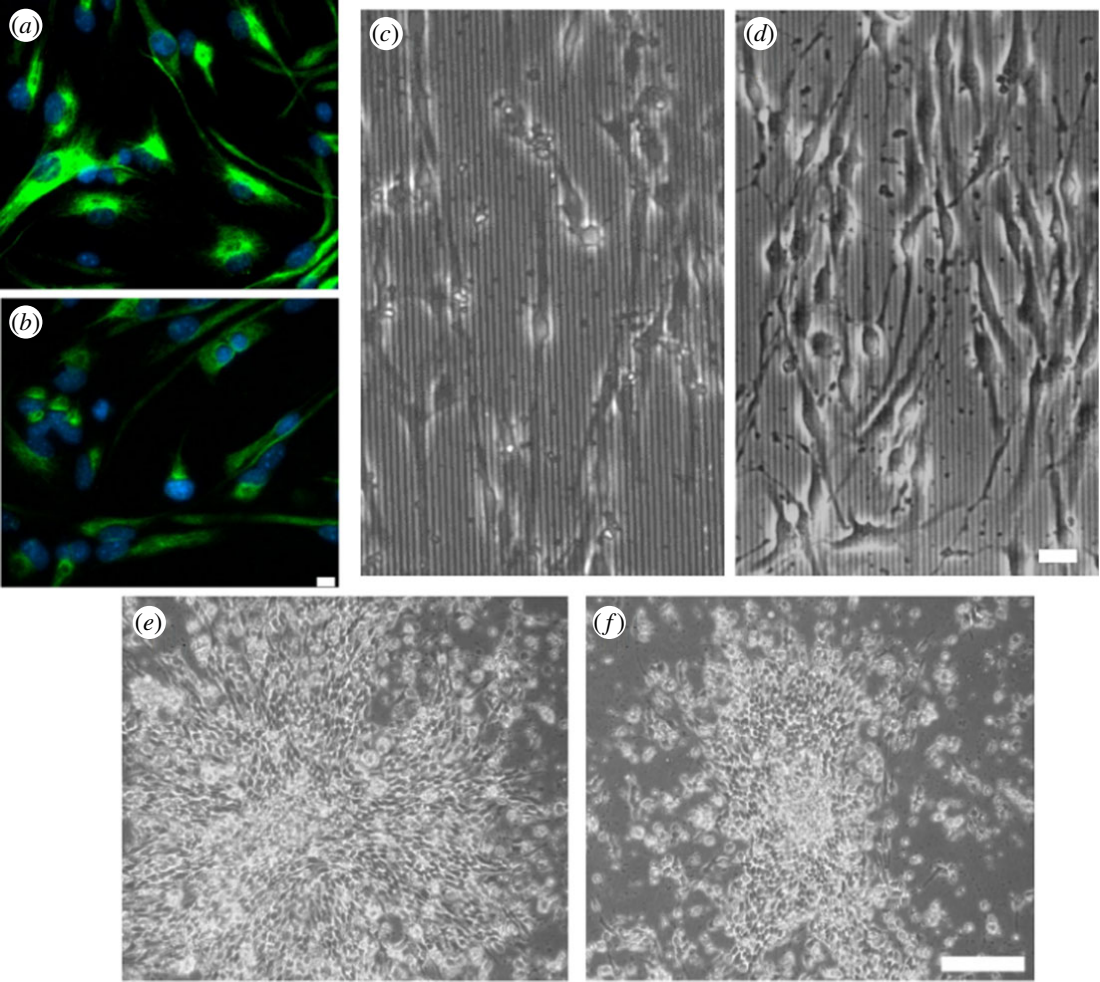
WT and *Pax6^−/−^* cell alignment on 2 µm grooves. (*a*,*b*) Nestin immunostaining for WT (*a*) and *Pax6^−/−^* (*b*) cells. Nuclei are stained with DAPI. (*c*,*d*) Representative image of WT (*c*) and *Pax6^−/−^* (*d*) cells plated on 2 µm grooves. (*e,f*) Neurospheres from WT embryos plated on flat quartz (*e*) and on 2 µm grooves (*f*). Scale bars: (*a*–*d*) 25 µm, (*e*,*f*) 500 µm.

**Figure 2.**
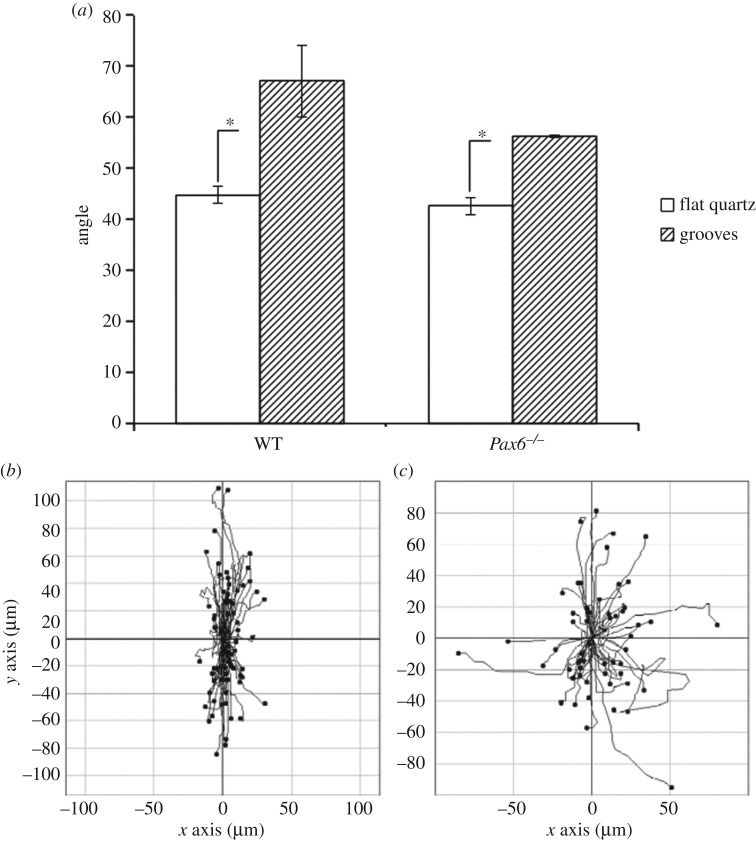
Migration alignment of cells on vertical grooves. (*a*) Average orientation angle for WT and *Pax6^−/−^* cells migrating on flat quartz or 2 µm grooves. Data are mean and standard deviation of three independent experiments. (*b*,*c*) Individual cell trajectories, plotted from the same origin, for WT (*b*) and *Pax6^−/−^* (*c*) cells. *p < 0.05.

Cells derived from WT and *Pax6^−/−^* mouse embryonic cortex were initially grown in suspension as neurospheres [18], and subsequently in adherent culture, where most cells expressed the neural precursor marker nestin (figure 1*a*,*b*). To subject cells to contact guidance cues, cells were then cultured on quartz slides with grooves of defined width and depth of 2 µm and 130 nm, respectively, which have previously been shown to induce marked cell alignment [10,11,19]. We observed extensive alignment on grooves as well, for both WT and *Pax6^−/−^* cells (figure 1*c*,*d*), even when cells were plated as undissociated neurospheres (figure 1*e*,*f*). To quantify the degree to which cells migrate parallel to grooves, we defined the orientation angle as the absolute value of the acute angle formed between grooves and a line connecting the first and last points of a cell trajectory, which takes values between 0° and 90°. For both WT and *Pax6^−/−^* cells plated in flat quartz, the average orientation angle was close to 45°, indicative of unbiased migration, whereas it was significantly higher in quartz with vertical grooves (figure 2*a*), indicative of cell trajectories highly aligned to grooves (figure 2*b*,*c*). Therefore, both WT and *Pax6^−/−^* cells displayed marked responses to contact guidance cues in the form of substratum nanogrooves.

In flat quartz, when we applied a physiological EF of 250 mV mm^−1^ [3], we observed a strong electrotactic response of both WT and *Pax6^−/−^* cells, both migrating towards the cathode with high values of directedness (see Material and methods), as previously observed for WT embryonic and adult neural stem cells [8,9].

In summary, when substratum nanogrooves and EFs were presented separately, both WT and *Pax6^−/−^* cells had marked responses to each guidance cue.

### 2.2. Response of cells exposed to grooves and electric fields simultaneously

**Figure 3.**
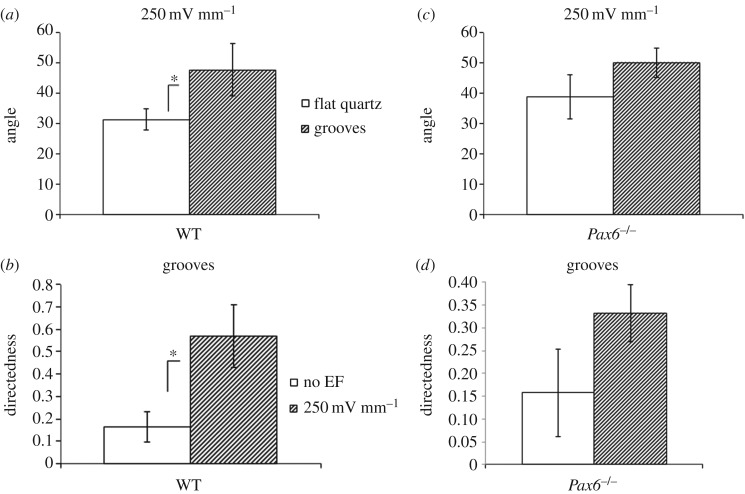
Average orientation angle and directedness of WT and *Pax6^−/−^* cells exposed simultaneously to vertical grooves and a horizontal EF. (*a*,*c*) Average orientation angle of WT (*a*) and of *Pax6^−/−^* (*c*) cells exposed to 250 mV mm^−1^ either on flat quartz or 2 µm grooves. (*b*,*d*) Directedness for WT (*b*) and *Pax6^−/−^* (*d*) cells plated on 2 µm grooves either in the absence of an EF or exposed to 250 mV mm^−1^. Data are mean and standard deviation of three independent experiments. *p < 0.05.

**Figure 4.**
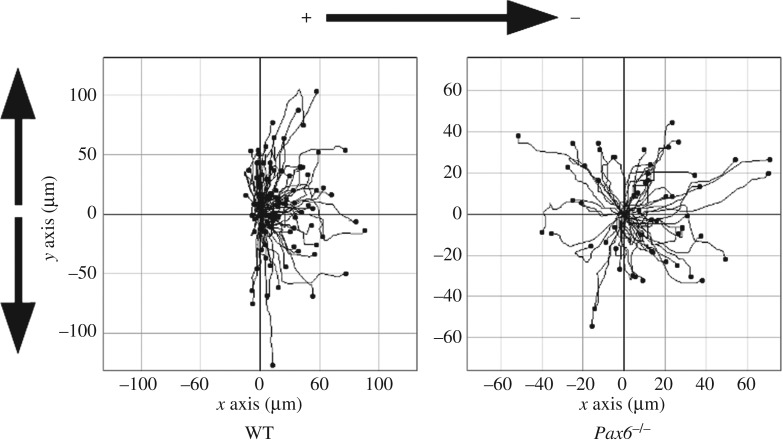
Individual cell trajectories, plotted from the same origin, of WT and *Pax6^−/−^* cells exposed simultaneously to vertical 2 µm grooves and a horizontal 250 mV mm^−1^ EF.

**Figure 5.**
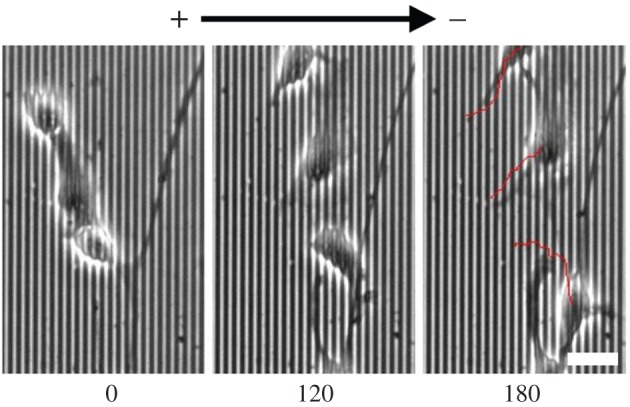
Example of migration of WT cells exposed simultaneously to 2 µm grooves and a 250 mV mm^−1^ EF perpendicular to each other. Each image in the sequence corresponds to the same region, at the indicated time (minutes), and the last image also shows the trajectory of each cell (see also the electronic supplementary material, video S1). Scale bar, 25 µm.

To expose cells simultaneously to diverging contact guidance and EF cues, we plated cells on vertical grooves and applied an EF with its vector oriented horizontally, so that both cues are perpendicular to each other. For WT cells, we observed that the orientation angle was significantly higher on grooves than on flat quartz when exposed to an EF, indicating that cells still responded to grooves when exposed simultaneously to a perpendicular EF (figure 3*a*). At the same time, directedness was significantly higher in WT cells exposed to both grooves and EFs compared to cells exposed to grooves alone (figure 3*b*), indicating that cells also retained an electrotactic response in the presence of grooves. Cell trajectories were oriented towards the cathode, but were also highly oblique, as shown in detail in figure 4 and exemplified in figure 5 (see also the electronic supplementary material, video S1). Therefore, WT neural cells had the capacity to integrate both guidance cues, moving in trajectories biased towards the cathode but at the same time significantly aligned to grooves.

**Figure 6.**
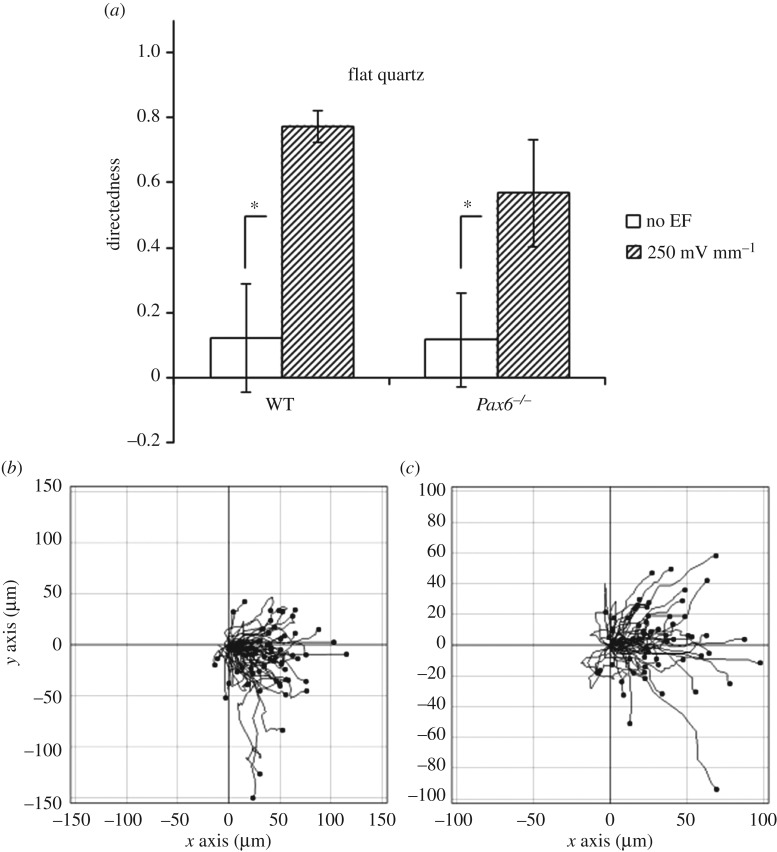
Electrotaxis of cells on flat quartz. (*a*) Directedness for WT and *Pax6^−/−^* cells on flat quartz either in the absence or the presence of a horizontal 250 mV mm^−1^ EF (cathode on the left). Data are mean and standard deviation of three independent experiments. (*b*,*c*) Individual cell trajectories, plotted from the same origin, for WT (*b*) and *Pax6^−/−^* (*c*) cells. *p < 0.05.

By contrast, *Pax6^−/−^* cells did not show a significantly higher orientation angle on grooves than on flat quartz when exposed to an EF (figure 3*c*), indicating an impaired ability to align their migration to grooves when exposed simultaneously to an EF. At the same time, even though *Pax6^−/−^* cells increased directedness when exposed to both grooves and EFs compared to grooves alone, this increase was not statistically significant (figure 3*d*, compare to figure 6). In agreement with these data, *Pax6^−/−^* cell trajectories showed clearly diminished cathodal and vertical biases compared to WT cells (figure 4).

In summary, while WT cells retained both cathodal and groove aligned biases when exposed simultaneously to grooves and EF, *Pax6^−/−^* cells were not able to respond significantly to both cues in combination. Therefore, absence of *Pax6* impairs the ability of cells derived from cortical neurospheres to integrate diverging guidance cues.

## 3. Discussion

In this study, we used primary cultures of WT and *Pax6^−/−^* cells derived from cortical neurospheres. When plated on grooved substrates, both WT and *Pax6^−/−^* cells showed alignment on grooves and contact guidance behaviour, and both displayed electrotactic responses on flat quartz when exposed to EFs. However, their behaviour when exposed to the two guidance cues in conflicting orientation differed: whereas WT cells displayed marked electrotactic responses on grooves and a significantly higher orientation angle than on flat quartz, *Pax6^−/−^* cells did not maintain either significant electrotaxis or a higher orientation angle on grooves, implying that the ability to integrate different guidance cues is impaired in cells lacking *Pax6* function.

These cells displayed remarkable responses to contact guidance cues, which led to the formation of ordered patterns *in vitro*, as illustrated by the alignment of cells and their trajectories on grooves and also by the migration of cells away from neurospheres predominantly along grooves, even though the high density of cells in the adhering neurospheres might have pushed cells to migrate in all directions (figure 1*f*). Moreover, our experiments have shown that these neurosphere-derived cells can integrate complex external cues that drive alignment and migration *in vitro*.

During Central nervous sytem (CNS) development *in vivo*, true neural progenitors (the stem cells) show polar alignment in the plane of the membrane but do not generally migrate radially, remaining in contact with the apical surface of the neural epithelium [20]. They divide to produce postmitotic, migratory precursors that perform complex migrations likely to be driven by both contact-mediated and electric cues [4,5]. It is likely that the migrations observed in this study are most analogous to these migrations of neural precursors *in vivo*, and hence relevant to architecture of the developing CNS. In regenerative experimental scenarios, neurosphere-derived neural progenitors can undergo long distance migrations in response to injury or inflammation [21], and the experiments described here contribute to our understanding of how they may do this, integrating contact mediated guidance cues with disturbance in ion flow caused by injury. *Pax6* is expressed in neural stem cells and is one of the genes required for the division/differentiation decision. It is possible that the differences observed between WT and *Pax6* mutant cells in the assays described here represent differences in the cell populations because of the requirement for *Pax6* in correctly specified neural stem cell behaviour [22].

Aside from its importance to CNS development and repair, the work described in this study contributes to the growing understanding of the ‘space perception’ of cells in terms of how they respond to their environment. Modern tools such as optogenetic techniques can be used in conjunction with experiments such as these to elucidate the cellular response to the environment [23].

WT cells exposed simultaneously to EFs and grooves oriented perpendicular to each other displayed behaviour consistent with integration of these guidance cues. They migrated to the cathode, as indicated by a significant increase of the directedness parameter after EF exposure, but at the same time maintained a trajectory oblique to the EF vector, as opposed to cells in flat quartz migrating more parallel to it, which is evidenced by the significantly higher orientation angle in grooves compared to flat quartz in the presence of EFs. This behaviour could be interpreted as the result of a sum of two vectorial guidance cues, the contact guidance vector pointing either up or down and the EF vector pointing towards the cathode, situated to the right. The resulting vector would determine an oblique trajectory directed overall to the cathode, as observed experimentally (figures 4 and 5). The precise orientation of such a vector would depend on the relative strength of the response of cells to grooves and EFs. Equal responses to both would be expected to give rise to a trajectory oriented with a 45° angle both to grooves and the EF vector.

Interestingly, the average orientation angle of WT cells on 2 µm grooves exposed to an EF of 250 mV mm^−1^ was 47.7°, with a standard deviation of 8.6°. This could be taken to indicate that a population of WT cells responds on average with relatively equal strength to the contact guidance cues imparted by 2 µm wide grooves and an EF of 250 mV mm^−1^. Variations in individual trajectories might reflect variations in the relative strength of the response to each guidance cue by individual cells.

This integrative response of WT cells is similar to that of corneal epithelial cells, the other cell type previously exposed to grooves and EFs oriented perpendicular to each other. Corneal epithelial cells also maintained contact guidance when exposed to EFs, while at the same time showing an overall migration towards the cathode [10]. Taken together, these results and ours support the notion that the integrative migratory responses we observed are probably a widespread property of motile cells.

*Pax6^−/−^* cells displayed contact guidance on grooves in the absence of EFs. They also showed a significant electrotactic response on flat quartz. However, on grooves, although directedness increased after EF exposure, its increase was not significant, and similarly, the orientation angle was not significantly higher on grooves than in flat quartz in EFs (figure 3*c*,*d*). Therefore, unlike WT cells, their *Pax6^−/−^* counterparts had an impaired ability to integrate EFs and contact guidance cues when presented in conflicting orientations.

*Pax6* is a transcription factor, whereas the cell orientation and migration decisions that we observe are made on rapid time-scales, probably in the absence of immediate transcriptional changes. However, *Pax6* controls the expression of a great number of genes, including many coding for cell adhesion molecules required for migratory responses and alignment [24]. Therefore, *Pax6* deficient cells failure to align or migrate properly when challenged could be conceivably caused by pre-existing deficiencies of the response pathways owing to loss of Pax6, rather than by defects in decision-making processes requiring Pax6-mediated changes in gene expression. For instance, the defects in migration of *Pax6^−/−^* neurons and neural progenitors, which have been observed *in vitro* and in chimerical embryos containing both WT and *Pax6^−/−^* cells, have been attributed to abnormal expression of cell-adhesion proteins [16], and also to altered cytoskeletal dynamics [17]. On the other hand, *Pax6* participates in conserved gene regulatory networks that control cell migration during development [25], and its absence could conceivably alter many different aspects of the migratory phenotype of cells. Our results suggest that *Pax6* is likely to be involved in fine-tuning precise migratory responses of cells that integrate multiple guidance cues in their microenvironment.

## 4. Material and methods

### 4.1. Mice

*Pax6^*Sey*-*Neu/+*^* mice, heterozygous for an inactivating mutation in *Pax6* that acts as a genetic null [26], were maintained on the CBA/Ca genetic background and crossed under Home Office (UK) licence in order to obtain homozygous, heterozygous and wild-type littermates. Genotypes were confirmed by PCR, as previously described [27]. WT mice on the CBA/genetic background were used to derive WT cells.

### 4.2. Cells derived from cortical neurospheres

Cerebral cortices from embryonic day 15.5 were chopped and filtered through a 40 µm pore size cell strainer, and the resulting cell suspensions were plated on uncoated 25 cm [2] flasks in DMEM/F-12 medium supplemented with N2 (both from Invitrogen, Paisley, UK) and 20 ng ml^−1^ FGF-2 and EGF (Peprotech, London, UK). Neurospheres formed after approximately one week, after which they were plated on poly-ornithine/laminin-coated plastic 25 cm [2] flasks to obtain adherent cultures.

### 4.3. Immunostaining

Cells were fixed with 4% paraformaldehyde, permeabilized with 0.2% Triton X-100 and incubated in blocking solution (1% bovine serum albumin in phosphate buffered saline (PBS)) for 30 min before incubation with mouse monoclonal anti-nestin antibody (1:200; Abcam, Cambridge, UK) at 4°C overnight. Cells were then incubated with fluorescein isothiocyanate-conjugated goat anti-mouse secondary antibodies (1:200; Jackson Immunoresearch, UK) at 37°C for 1 h, and mounted in Vectashield mounting medium with 4′,6-diamidino-2-phenylindole (DAPI) (Vector Laboratories, Peterborough, UK).

### 4.4. Cell culture on substratum nanogrooves

Grooved substrata were prepared in a previous study by the Department of Electronics and Engineering, Glasgow University, using electron beam lithography on fused quartz microscope slides [19]. We used slides with parallel grooves, 130 nm deep and 2 µm wide, which also had areas of flat quartz used as a flat substratum control. Slides were sterilized in concentrated nitric acid, rinsed extensively in sterile PBS and coated with poly-ornithine/laminin. Cells were then plated on the coated slides and maintained overnight before experiments.

### 4.5. Electric field application

EFs were applied to cells adhered to flat quartz or grooves as previously described [10]. Briefly, the quartz slides were inverted (cell side down) over a chamber made by gluing two parallel strips of coverslip to the bottom of a culture dish. Then, agar-salt bridges (filled with Steinberg's solution gelled with 2% agar) were used to connect silver–silver chloride electrodes in beakers of Steinberg's solution to reservoirs of culture medium at either side of the chamber. An EF of 250 mV mm^−1^ was applied, and the pH was kept stable by adding 4-(2-hydroxyethyl)-1-piperazineethanesulfonic acid (HEPES) to the medium (25 mM final concentration).

### 4.6. Time-lapse imaging and analysis

Time-lapse imaging was performed at 37°C using a Zeiss Axiovert 100 microscope equipped with a motorized stage (Universal Imaging Corporation, Downingtown, PA, USA) a digital camera (PDMI-2; Medical System Corp., Greenvale, NY, USA) and the MetaMorph 6.1 imaging system. Phase-contrast images were obtained every 5 min for 2–3 h. To quantify migration alignment to grooves, the absolute value of the acute angle between the cell displacement vector (connecting the first and last point of a cell trajectory) and the grooves was measured. This was termed the orientation angle, and its value is independent of the direction of cell migration. The average of the orientation angle was calculated for a migratory cell population for each experiment. To quantify electrotaxis, we calculated the directedness parameter, as previously described [8], which is defined as the cell population average of cos *θ*, where *θ* is the angle between the EF vector and the cell displacement vector. A directedness value close to 1 or −1 indicates strong cell migration towards the cathode or anode, respectively, whereas a value close to 0 indicates absence of electrotaxis. For each experiment, between 50 and 100 different cell trajectories were analysed. Three independent experiments were performed for each condition. Trajectory data and a summary of directedness and angle values are available from the Dryad Digital Repository [28]. Comparisons between different experimental conditions were made using an unpaired two-tailed Student's *t*-test.

## Data accessibility

Cell trajectories data and a summary of directedness and angle values are deposited at Dryad: http://dx.doi.org/10.5061/dryad.53512 [28].
